# Fetal-type posterior communicating artery increases hemodynamic stress in posterior communicating artery bifurcation aneurysms: a CFD-based analysis

**DOI:** 10.1007/s00234-025-03785-w

**Published:** 2025-09-20

**Authors:** Roland Schwab, Rebecca Janiszewski, Erelle Fuchs, Maximilian Thormann, Belal Neyazi, Vanessa Magdalena Swiatek, I. Erol Sandalcioglu, Philipp Berg, Daniel Behme, Samuel Voß, Janneck Stahl

**Affiliations:** 1 University Clinic for Neuroradiology, University Hospital Magdeburg, Magdeburg, Germany; 2 Research Campus STIMULATE, Otto-von-Guericke University Magdeburg, Magdeburg, Germany; 3 Department of Nuclear Medicine, Charité - University Medicine Berlin, Berlin, Germany; 4 Department of Radiology and Nuclear Medicine, University Hospital Magdeburg, Magdeburg, Germany; 5 Department of Neurosurgery, Otto-von-Guericke University Magdeburg, Magdeburg, Germany; 6 Research Campus STIMULATE, Otto-von-Guericke University Magdeburg, Magdeburg, Germany; 7 Department of Medical Engineering, Otto-von-Guericke University Magdeburg, Magdeburg, Germany; 8 Department of Fluid Dynamics and Technical Flows, Otto-von-Guericke University Magdeburg, Magdeburg, Germany

**Keywords:** Aneurysm, Endovascular, Hemodynamics, PCOM

## Abstract

**Backround:**

The flow characteristics of bifurcation aneurysms in the posterior communicating artery (PCOM) have rarely been studied. The likelihood of a complete PCOM aneurysm occlusion after endovascular treatment is reduced with the presence of a fetal posterior communicating artery (fPCOM). As a result, anatomical variations in PCOM aneurysms represent a major challenge for the endovascular treatment. This study addresses hemodynamic variations in PCOM aneurysms of either fetal or adult type.

**Methods:**

3D-DSA data of 14 patients with bifurcation aneurysms located in the PCOM junction were collected. Nine patients presented with a fPCOM and five patients an adult PCOM (aPCOM). Patient-specific 3D models containing at least one bifurcation distal the aneurysm in the anterior circulation as well as the PCOM itself were extracted using image-based blood flow simulations. Seven hemodynamic parameters were calculated for all aneurysm models to characterize the intra-aneurysmal blood flow. The PCOM outflow was artificially varied to represent both fPCOM and aPCOM conditions for each model resulting in 28 simulations.

**Results:**

Fetal‑type PCOM showed higher intra‑aneurysmal mean velocity (median 0.09 vs. 0.06 m/s), maximum velocity (0.17 vs. 0.14 m/s), averaged wall shear stress (1.67 vs. 1.27 Pa), neck inflow rate (40.9 vs. 22.4 ml/min), and inflow concentration index (0.56 vs. 0.40), with lower pulsatility index (1.73 vs. 1.89). Those differences were significant whereas mean oscillatory shear index did not differ significantly.

**Conclusion:**

The presence of anatomical variations affects the hemodynamic parameters of PCOM bifurcation aneurysms. In particular, the presence of an fPCOM has an unfavorable effect on the intra-aneurysmal flow dynamics.

**Supplementary Information:**

The online version contains supplementary material available at 10.1007/s00234-025-03785-w.

## Introduction

Posterior communicating artery (PCOM) aneurysms are among the most common intracranial aneurysms, accounting for roughly 15–25% of all cases and about half of internal carotid artery aneurysms [1, 2]. Clinically, they are significant causes of subarachnoid hemorrhaging [1–4]. Modern management of PCOM aneurysms has increasingly favored endovascular approaches. Endovascular coil embolization is a well-established first-line treatment for PCOM aneurysms [1, 5]. In recent years, flow-diverting stents have also emerged as a treatment option for complex or recurrent PCOM aneurysms, aiming to redirect blood flow and promote intra-aneurysmal thrombosis [6, 7]. Despite advances in technique, PCOM aneurysms in particular have shown high recurrence rates relative to other common aneurysm locations [8, 9]. Large series report that approximately one-third of PCOM aneurysms develop radiographic recanalization after coiling on follow-up imaging [8]. This rate is among the highest for aneurysms and necessitates diligent surveillance. Established risk factors for post-treatment recurrence include larger aneurysm size, incomplete initial occlusion, and rupture at presentation [10–12]. In addition, anatomical and hemodynamic factors unique to the PCOM may influence outcomes. One such factor is the fetal-type PCOM (fPCOM), an anatomical variant present in around 20% of patients [10]. With the fetal variation, the PCOM is enlarged and serves as the principal supply to the posterior cerebral artery, fundamentally altering flow dynamics. This variant predisposes poorer aneurysm occlusion rates [10]. The fetal posterior circulation variant is widely regarded as an important consideration in PCOM aneurysm management due to the hemodynamic load it imposes. The influence of a fPCOM is also evident in the context of flow diversion therapy [6, 7]. These findings underscore that PCOM aneurysms with a fetal-type anatomy pose a particular challenge, with both anatomical and hemodynamic factors contributing to higher recurrence rates and less favorable occlusion outcomes [6, 8]. In recent years, computational fluid dynamics (CFD) has become an established research tool for assessing the hemodynamic stability of intracranial aneurysms. However, the hemodynamics of PCOM aneurysms, particularly in relation to different anatomical variants, have been rarely investigated using CFD approaches. Recently, Huang et al. and Hu et al. analyzed morphological and hemodynamic characteristics in ruptured and unruptured PCOM aneurysms, but did not specifically include fPCOM configurations in their analyses [13, 14]. To date, Tanaka et al. published the only systematical study comparing fPCOM and adult PCOM (aPCOM) aneurysms [10]. Building on this, the present study extends numerical analyses by including both anatomical variants and introducing controlled modifications of PCOM outflow to simulate fetal- and adult-type flow conditions. This approach enables a more detailed evaluation of hemodynamic influences on aneurysm stability, providing valuable insights into the role of anatomical and flow-related factors in PCOM aneurysm management. Understanding the interaction of these factors is critical for guiding treatment selection and improving long-term results. Accordingly, this study focuses on how a fPCOM configuration impacts intra-aneurysmal flow dynamics, with the goal of understanding the clinical implications for this common, yet challenging, aneurysm subtype.

## Methods

### Patient data

Data was collected from patients treated for aneurysms in the PCOM junction at the Magdeburg University Hospital between 2003 and 2023. A total of 108 patients were initially screened. For inclusion, patients were required to have a PCOM bifurcation aneurysm and undergone pre-treatment digital subtraction angiography (DSA). This includes three-dimensional rotational DSA (3D-DSA) of the internal carotid artery (ICA) at least from the cavernous segment till the proximal M2 Segment of the middle cerebral artery (MCA) and A2 Segment of the anterior cerebral artery (ACA), with imaging quality sufficient to enable high-fidelity reconstruction of a three-dimensional vascular model. 3D-DSA images were acquired with a biplane angiographic unit (Artis Q, Siemens Healthineers GmbH, Germany. The isotropic edge length of the voxels is 0.28 mm. The reconstructed volume has a width and height of 142.91 mm each at a uniform resolution of 512 voxels in x- and y-direction. Criteria for high image quality included complete visualization of the ICA, inclusion of all relevant arterial branches, absence of discontinuities in vessel structures, and clear delineation of the aneurysm from adjacent vasculature. A total of 41 aneurysms were excluded due to their sidewall location, 29 due to the absence of 3D-DSA, 24 owing to incomplete depiction of adjacent vascular segments, and 14 because of insufficient image quality. The final study cohort consisted of 14 aneurysms. Based on vascular anatomy, patients were categorized into two groups. Five patients exhibited an aPCOM configuration, and nine patients exhibited a fPCOM configuration. A fetal configuration was defined as a PCOM diameter at least twice that of the P1 segment of the posterior cerebral artery (PCA) [6]. The selection of aneurysms was based exclusively on 3D-DSA. The assessment of the anterior and posterior circulation, particularly the configuration of the PCOM, was additionally performed using pre-interventional imaging modalities, including 2D-DSA, computed tomography angiography, and magnetic resonance angiography.

### 3D model extraction

Using the image processing program MeVisLab v. 3.7.2 (MeVis Medical Solutions AG, Bremen, Germany), an initial 3D surface model was segmented from the 3D DSA image data using a threshold-based method. This initial segmentation was converted into a triangulated 3D surface grid using the Marching Cubes algorithm.

After the initial segmentation, artifacts appeared on the surface mesh, which were removed using Blender Version v. 4.0 (Blender Foundation, Amsterdam, Netherlands). The vascular tree was reduced to a uniform region of interest for all cases to ensure consistency. The extracted models included the ICA starting from the C2 segment, the PCOM and the PCA up to the P2 segment. Distally, the bifurcations into the ACA and MCA were preserved up to the A1 and M1 segments, respectively. Smaller side branches, which were expected to have negligible hemodynamic influence on the main vessel segments, were removed using Boolean subtraction cuts. Separations of vessel fusions as well as the filling of vessel interruptions were conducted. Furthermore, local smoothing operations were performed to remove non-morphological surface irregularities and sharp edges using the sculpting tool integrated in Blender. This manipulation was conducted using a small region of interest for the adjustment of obvious artificial structures without changing the vascular morphology. Lastly, a sufficient extrusion of the inflow and outflow cross-sections to six times the corresponding vessel diameter in accordance with existing recommendations was conducted [15].

## Image-based blood flow simulation

For the prediction of the blood flow in the extracted geometries the finite-volume solver StarCCM + v17.06 (Siemens PLM Software Inc., Plano, TX, USA) was used. Previously, spatial discretization was conducted using polyhedral cells with a base size of 0.07 mm. Additionally, five layers of prism cells was used to resolve the near-wall flow [16]. This resulted in a total number of finite volume cells ranging from 2.4 to 5.1 million. A grid dependency test confirmed that increasing the resolution significantly led to variations of less than 3% in the results. Due to the absence of patient-specific flow measurements, an averaged time-dependent inflow waveform from Durka et al. [17] was applied and scaled to the local vessel diameter. For the outflow boundary conditions, an advanced in-house flow-splitting approach was applied to obtain realistic outflow ratios based on the respective vessel morphology. First, a centerline along the vascular tree was computed. The centerline was then divided into subsections at each bifurcation in order to capture the flow division into the corresponding daughter branches. Unlike radius-based simplifications, this approach calculates the anatomical vessel cross-sections directly. Based on these cross-sectional areas, realistic flow distributions were derived to represent physiologically plausible outflow values with respect to the actual patient-specific vessel shape [18]. The creation of the opposing supply configuration was done by varying the outflow pathway of the PCOM and the subsequent bifurcation. Since no reference values are currently available in the literature that quantify flow through fetal or adult configurations, the outflow variation was carried out empirically. For this purpose, the percentage-based splitting ratios were adjusted such that a mean PCOM flow of 4–20 ml/min represented the adult configuration, while a mean PCOM flow of 20–82 ml/min represented the fetal configuration. A minimum difference of at least 13 ml/min and a mean difference of 37 ml/min in outflow were maintained to ensure sufficient deviation between the two configurations. Thus, the outflow was systematically adjusted via modified splitting ratios to establish a distinct hemodynamic difference between the two variants. This resulted in 28 simulation setups representing the initial as well as artificially induced modulated PCOM outflow scenario for each case. For all configurations, rigid and no-slip wall conditions are applied. Blood was treated as an incompressible (ρ = 1055 kg/m^3^) and non-Newtonian fluid (Carreau-Yasuda model parameters were taken from Formaggia et al. [19] with the assumption of laminar flow conditions. Two cardiac cycles were simulated. The first was used to reduce occurring initialization effects. The second was used for the subsequent hemodynamic analysis.

## Hemodynamic parameters

The averaged wall shear stress (AWSS) is defined as the mean tangential shear stress on the vessel lumen, calculated over the duration of one cardiac cycle. The mean oscillatory shear index (OSI_mean_) characterizes the directional changes in shear stress during a cardiac cycle. The mean velocity (v_mean_) and the maximum velocity (v_max_) are utilized to describe the mean and maximum velocity, respectively, within the aneurysm. The neck inflow rate (NIR) is a metric used to describe the blood flow into the aneurysm, while the pulsatory index (PI) is a measure of the pulsatility of the blood flow entering the aneurysm during systole and diastole [20]. The inflow concentration index (ICI) is calculated to quantify the concentration of the inflowing blood relative to the total flow in the parent vessel [21]. The aneurysm neck curve was generated using an in-house developed automated tool [22]. Based on this neck curve, the aneurysm sac was separated from the parent vessel, enabling a consistent and reproducible anatomical definition of the aneurysm ostium. This separation subsequently allowed for automated calculation of hemodynamic parameters within the aneurysm sac, at the ostium or the respective parent vessel.

### Statistical analysis

The statistical analysis was conducted using IBM SPSS Statistics (Version 29.0.2.0). The significance level was set to α = 0.05. The sample consisted of *n* = 14 aneurysms, each evaluated under both the aPCOM and fPCOM condition, resulting in paired measurements. Accordingly, a total of 28 values were obtained for each parameter, with the data representing dependent samples. The calculation of descriptive statistics (mean, median, and standard deviation) was conducted for each parameter. The normality assumption was tested using the Shapiro-Wilk test. Given the statistical significance of the result (*p* < 0.05), the assumption of a normal distribution was rejected. Consequently, the Mann-Whitney-U-Test for the comparison of the unpaired original anatomical cohort and the Wilcoxon signed-rank test for the paired extended cohort was applied as a non-parametric procedure for significance testing. To account for multiple comparsion, the Holm–Bonferroni correction was applied, and the corresponding adjusted p-values are reported. A post-hoc power analysis was conducted using G*Power (Version 3.1) to determine the minimum effect size detectable with the given sample size. Statistical power is conventionally set at 0.80 (β = 0.20), with an α-level of 0.05. Under these parameters, the analysis indicated that the smallest reliably detectable effect size was 0.72.

## Results

### Qualitative results

**Fig. 1:**
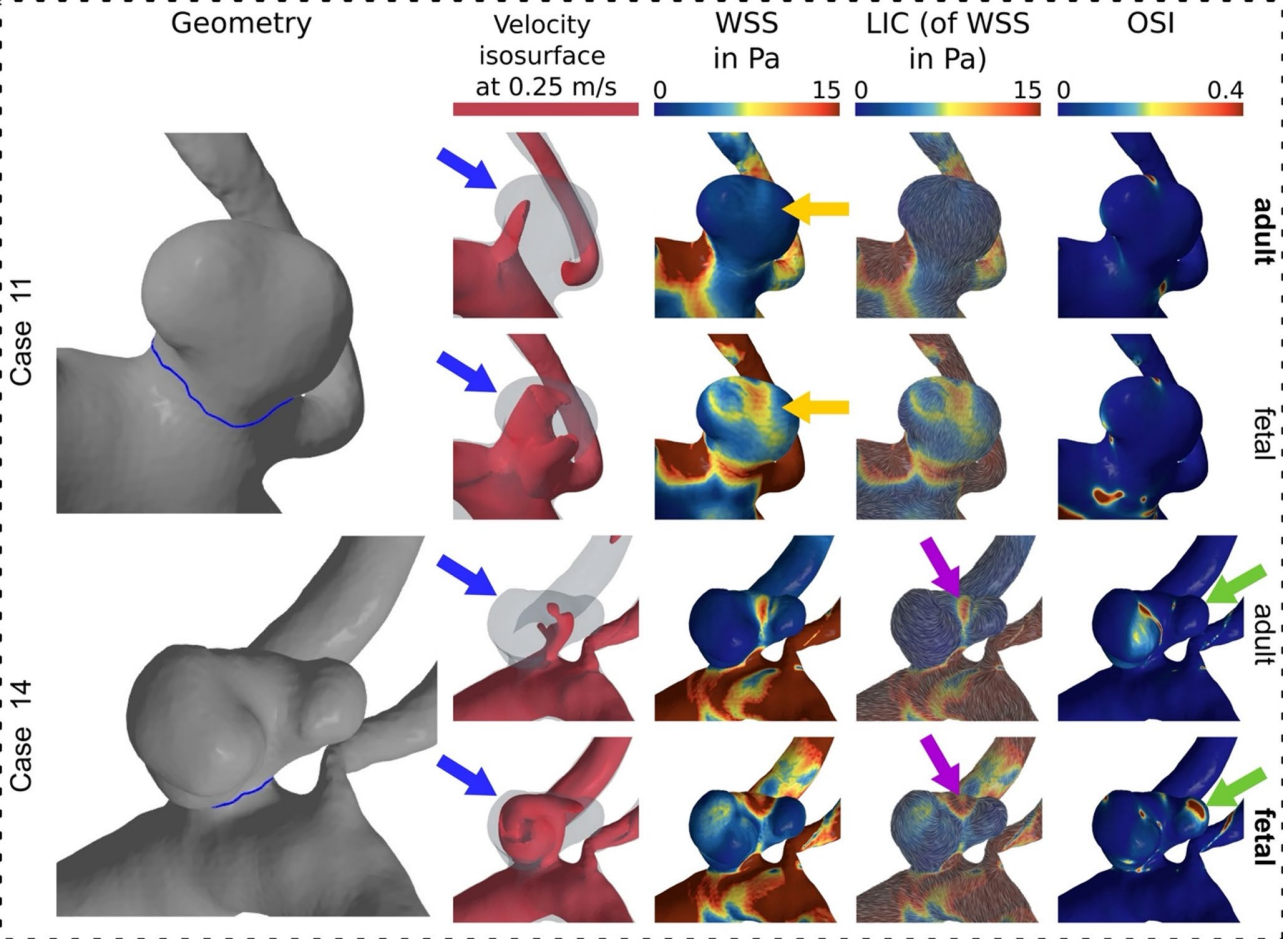
Qualitative results of two representative cases of a patient-specific fetal and adult configuration (bold font) as well as the artificially created opposite outflow scenario (regular). The first column shows the 3D geometry of the aneurysm with the neck curve in blue. Column two to five shows temporal averaged hemodynamic results: Velocity isosurface, wall shear stress (WSS), WSS visualized via line integral convolution (WSS LIC) and oscillatory shear index (OSI). Relevant changes are marked with colored arrows

In addition to the quantitative evaluation, a qualitative analysis was performed to visualize and better interpret the underlying flow patterns. Figure 1 illustrates representative cases in which the fPCOM and aPCOM variants are shown side by side for direct comparison (see supplementary material for all 14 cases). For each configuration, the following properties are displayed: Geometry, iso-surface of flow velocity (threshold ≥ 0,25 m/s), wall shear stress (WSS), WSS visualized via line integral convolution (WSS LIC, represents velocity vector field by integrating noise along streamlines, revealing directional features) and OSI.

In both configurations, the inflow into the aneurysm was frequently observed at velocities above 0.25 m/s. In the fetal configuration, however, this velocity threshold value was exceeded in a larger proportion of the volume. In addition, increased WSS was observed in the areas with increased velocity for both representative cases. It is particularly noticeable in case 11 that there was a strong global increase of the WSS in the aneurysm. However, in case 14 there was also a more pronounced spatial distribution of the high magnitude in the center of the dome over the entire surface of the aneurysm dome. The WSS LIC showed different patterns in the fetal variant as compared to the adult variant in particular for case 14. As a result, variations were observed not only in the magnitude of the values but also in their local distribution. Furthermore, the OSI showed a different spatial distribution within the aneurysm in the two configurations especially for case 14, whereas it was quite stable in case 11. The exemplary cases are shown in Fig. 1 and the qualitative results for all 14 cases are provided in the Supplementary Figs. 1–4.

## Quantitative results

**Fig. 2:**
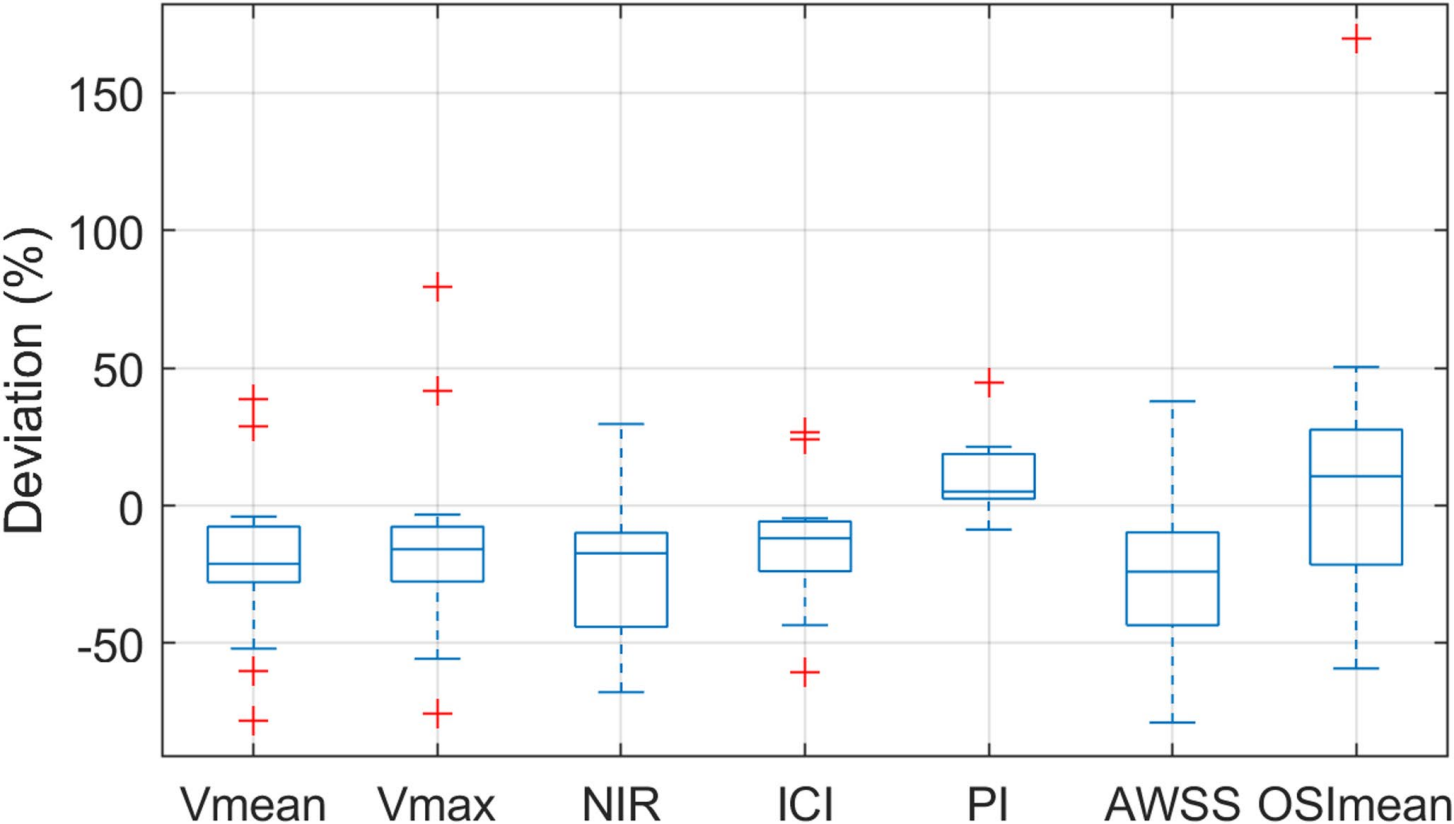
Boxplots showing the deviation from each fetal configuration to the corresponding adult configuration for the following hemodynamic parameters: v_mean,_ mean velocity; v_max,_ maximum velocity; NIR, neck inflow rate; ICI, inflow concentration index; PI, pulsatory index; AWSS, averaged wall shear stress; OSI_mean,_ mean oscillatory shear index

Table 1 shows an overview of the hemodynamic results, comparing the fetal flow variant with the normal flow variant for each patient. Overall, v_mean_ and v_max_ exhibited higher values in the fetal configuration in almost all cases, a trend that was also observed for AWSS, NIR, and ICI. The most pronounced differences in these parameters were seen in case 5. In contrast, cases 2 and 12 deviated from this pattern, showing higher values in the adult configuration. Regarding PI, most cases presented slightly higher values in the adult configuration, except for cases 1, 4 and 9. For OSI_mean_, both increasing and decreasing behaviors were observed, without a clear pattern related to outflow modulation. Additionally, no consistent relationship could be identified between the magnitude of outflow variation and the extent of hemodynamic parameter changes. Deviations from each fetal configuration to the corresponding adult configuration are presented in Fig. 2. Three hemodynamic parameters (v_mean_, v_max_ and ICI) are consistently lower in the adult configuration except for two outliers. NIR, AWSS and PI also indicate a clear tendency. OSI_mean_, however, no consistent pattern is observed, with deviations in both directions. Furthermore, Table 2 shows the descriptive statistics and the results of the Mann-Whitney-U-Test for the comparison of the unpaired original anatomical cohort and the Wilcoxon signed-rank test for the extended paired cohort of all evaluated parameters. In contrast to the original unpaired cohort, in which no statistically significant differences were detected, the extended paired cohort demonstrated significant differences in several hemodynamic parameters between the two anatomical variants. The fPCOM showed significantly higher values compared to the aPCOM. Specifically, the median of the v_mean_ exhibited a significant elevation in the fPCOM (z = 2.79; *p* = 0.024; *r* = 0.75), as well as the v_max_ (z = 2.54; *p* = 0.024; *r* = 0.68). In addition, both the NIR (z = 3.05; *p* = 0.014; *r* = 0.81) and the ICI (z = 2.92; *p* = 0.024; *r* = 0.78) were significantly higher in the fPCOM group. The AWSS also showed significantly higher values in fPCOM compared to aPCOM (z = 2.86, *p* = 0.024, *r* = 0.76). In contrast, the PI was significantly higher in the aPCOM variant (z = −2.67; *p* = 0.024; *r* = −0.71). For the OSI_mean_, no statistically significant difference was found (z = −0.66; *p* = 0.51; *r* = −0.18). However, a reversed trend was observed, with OSI_mean_ values in the aPCOM tending to be higher than those in the fPCOM.

**Table 1:** Quantitative comparison of the calculated hemodynamics for the fetal as well as the adult configuration of each case. The patient-specific PCOM outflow variant is shown in bold and the artificial outflow variation in standard text. In addition to the absolute values, the relative deviation (dev.) between the two configurations is presented

Case	PCOM outflow (ml/min)	Configuration	v_mean_ (m/s)	v_max_ (m/s)	NIR (ml/min)	ICI (-)	PI (-)	AWSS (Pa)	OSI_mean_ (-)
1	37	**fetal**	0.095	0.192	51.8	0.58	1.65	2.16	0.022
	9	adult	0.069	0.139	42.9	0.46	1.63	1.52	0.024
		*dev.*	*−28%*	*−28%*	*−17%*	*−20%*	*−2%*	*−30%*	*10%*
2	50	fetal	0.028	0.064	16.0	0.25	1.92	0.51	0.093
	18	**adult**	0.039	0.090	20.8	0.31	2.00	0.71	0.038
		*dev.*	*39%*	*42%*	*30%*	*27%*	*4%*	*38%*	*−59%*
3	39	fetal	0.042	0.102	21.6	0.35	2.20	0.89	0.029
	5	**adult**	0.039	0.099	15.9	0.31	2.34	0.70	0.033
		*dev.*	*−8%*	*−3%*	*−26%*	*−11%*	*7%*	*−22%*	*11%*
4	20	**fetal**	0.107	0.178	159.8	2.43	1.16	1.69	0.054
	7	adult	0.102	0.162	153.6	2.29	1.05	1.61	0.050
		*dev.*	*−4%*	*−9%*	*−4%*	*−6%*	*−9%*	*−5%*	*−7%*
5	57	**fetal**	0.038	0.091	24.6	0.29	2.12	0.91	0.035
	18	adult	0.008	0.022	7.9	0.12	2.53	0.19	0.094
		*dev.*	*−78%*	*−76%*	*−68%*	*−60%*	*19%*	*−79%*	*170%*
6	32	**fetal**	0.086	0.169	84.2	1.06	1.52	1.36	0.028
	12	adult	0.079	0.156	75.6	1.01	1.55	1.23	0.036
		*dev.*	*−9%*	*−8%*	*−10%*	*−5%*	*2%*	*−10%*	*28%*
7	85	**fetal**	0.247	0.488	115.5	1.26	1.51	6.82	0.016
	20	adult	0.213	0.433	97.8	1.11	1.60	6.12	0.020
		*dev.*	*−14%*	*−11%*	*−15%*	*−12%*	*6%*	*−10%*	*24%*
8	32	**fetal**	0.072	0.161	28.5	0.56	1.90	1.64	0.021
	4	adult	0.059	0.132	24.0	0.45	1.96	1.41	0.019
		*dev.*	*−19%*	*−18%*	*−16%*	*−21%*	*3%*	*−14%*	*−7%*
9	82	fetal	0.256	0.553	51.6	0.30	1.79	11.10	0.048
	19	**adult**	0.187	0.406	38.1	0.27	1.79	6.30	0.071
		*dev.*	*−27%*	*−27%*	*−26%*	*−11%*	*0%*	*−43%*	*48%*
10	32	fetal	0.024	0.048	25.2	0.56	1.66	0.38	0.027
	6	**adult**	0.019	0.042	14.0	0.43	2.01	0.25	0.031
		*dev.*	*−24%*	*−14%*	*−44%*	*−24%*	*21%*	*−34%*	*15%*
11	46	fetal	0.132	0.288	39.1	0.66	1.79	3.14	0.015
	8	**adult**	0.063	0.140	18.8	0.37	1.87	1.32	0.011
		*dev.*	*−52%*	*−51%*	*−52%*	*−43%*	*4%*	*−58%*	*−25%*
12	54	**fetal**	0.014	0.047	2.4	0.02	3.13	0.30	0.030
	15	adult	0.018	0.085	2.7	0.03	4.53	0.40	0.045
		*dev.*	*29%*	*79%*	*15%*	*24%*	*45%*	*34%*	*50%*
13	30	**fetal**	0.097	0.200	52.3	1.23	1.67	1.83	0.019
	6	adult	0.072	0.157	43.1	1.14	1.83	1.36	0.014
		*dev.*	*−25%*	*−21%*	*−18%*	*−7%*	*10%*	*−26%*	*−26%*
14	64	**fetal**	0.139	0.283	42.7	0.49	1.62	2.89	0.019
	15	adult	0.056	0.125	19.3	0.29	1.93	1.04	0.015
		*dev.*	*−60%*	*−56%*	*−55%*	*−41%*	*19%*	*−64%*	*−21%*

PCOM, posterior communicating artery; v_mean,_ mean velocity; v_max,_ maximum velocity; NIR, neck inflow rate; ICI, inflow concentration index; PI, pulsatory index; AWSS, averaged wall shear stress; OSI_mean,_ mean oscillatory shear index

**Table 2:** Descriptive statistics and differences of the median values of hemodynamic parameters between the fPCOM and aPCOM aneurysms in the original unpaired and extended paired cohort. Statistically significant p-values are marked with an asterisk

Parameter	Median fPCOM	95% CI	Median aPCOM	95% CI	z-value	*p*-value unadjusted	*p*-value adjusted	Effect size *r*
Original cohort (*N* = 14)								
v_mean_ (m/s)	0.10	[0.07; 0.11]	0.40	[0.04; 0.06]	−0.33	0.74	> 0.99	−0.09
v_max_ (m/s)	0.18	[0.16; 0.20]	0.10	[0.09; 0.14]	−0.20	0.84	> 0.99	−0.05
NIR (ml/min)	51.74	[28.48; 84.28]	18.74	[15.91; 20.81]	−1.53	0.13	0.91	−0.41
ICI (-)	0.58	[0.39; 1.25]	0.31	[0.31; 0.37]	−1.27	0.21	> 0.99	−0.34
PI (-)	1.65	[1.52; 1.90]	2	[1.93; 2.01]	−1.27	0.21	> 0.99	−0.34
AWSS (Pa)	1.69	[1.36; 2.16]	0.71	[0.69; 1.32]	−0.33	0.74	> 0.99	−0.09
OSI_mean_ (-)	0.02	[0.02; 0.03]	0.03	[0.03; 0.04]	−0.74	0.46	> 0.99	−0.20
Paired cohort (*N* = 28)								
v_mean_ (m/s)	0.09	[0.04; 0.11]	0.06	[0.04; 0.07]	2.79	0.005*	0.024*	0.75
v_max_ (m/s)	0.17	[0.10; 0.24]	0.14	[0.10; 0.16]	2.54	0.011*	0.024*	0.68
NIR (ml/min)	40.9	[25.19; 51.98]	22.4	[15,92; 49.99]	3.05	0.002*	0.014*	0.81
ICI (-)	0.56	[0.30; 1.06]	0.40	[0.29; 1.01]	2.92	0.004*	0.024*	0.78
PI (-)	1.73	[1.64; 1.91]	1.89	[1.73; 2.00]	−2.67	0.008*	0.024*	−0.71
AWSS (Pa)	1.67	[0.90; 2.53]	1.27	[0.71; 1.42]	2.86	0.004*	0.024*	0.76
OSI_mean_ (-)	0.03	[0.02; 0.03]	0.03	[0.02; 0.04]	−0.66	0.51	0.51	−0.18

*fPCOM*, fetal posterior communicating artery; *aPCOM* adult posterior communicating artery; *CI* confidence interval; *v*_*mean*,_ mean velocity; *v*_*max*,_ maximum velocity; *NIR* neck inflow rate; *ICI* inflow concentration index; *PI* pulsatory index; *AWSS* averaged wall shear stress; *OSI*_*mean*,_ mean oscillatory shear index

## Discussion

Retrospective studies showed that the morphology of vessels and aneurysms, including the diameter of the PCOM, as well as the aneurysm size and neck width, significant influences the local hemodynamics and can increase the risk of recurrence [23]. The preliminary analysis of the original unpaired group revealed slight trends without statistically significant differences in the hemodynamic parameters. We consider this to be mainly attributable to heterogeneity in aneurysm morphology, volume, size, neck width, PCOM exit location, and caliber of the ICA branches, in a limited sample size. The exact mechanisms underlying hemodynamic differences in PCOM aneurysms remains insufficiently explored. To enhance the understanding, the presented study investigated how variations in PCOM flow alone influences the hemodynamic characteristics within the aneurysm. By modulating the PCOM flow conditions, fetal and adult configurations were simulated within identical anatomical models. This approach enables the assessment of the isolated hemodynamic effects on intra-aneurysmal dynamics. The two configurations used in the comparison shared the aforementioned identical aneurysm specifications. As a result, these findings identify PCOM flow as an independent factor influencing the hemodynamic profile of PCOM aneurysms. The performed CFD simulations indicate that PCOM aneurysms in cases with fPCOM configuration experience markedly different flow patterns compared to those with an aPCOM. Notably, the fPCOM group exhibited higher intra-aneurysmal flow velocity and elevated WSS, along with a more concentrated inflow jet and reduced PI within the sac. These findings align with prior hemodynamic studies. Chung et al. investigated 313 PCOM aneurysms, including their angioarchitecture, without differentiating between aPCOM and fPCOM configurations. They reported that ruptured PCOM aneurysms tend to exhibit strong, concentrated inflow jets and localized regions of elevated WSS, along with more complex and oscillatory flow patterns compared to aneurysms in a sidewall configuration [24]. Similarly, a recent CFD analysis by Tanaka et al. found that fPCOM aneurysms exhibited significantly higher intra-aneurysmal flow velocity and WSS compared to aPCOM aneurysms [10]. In our study, the reduced pulsatility of flow in fetal configurations suggests a more continuous, siphoning blood supply from the ICA, whereas in a normal configuration the aPCOM flow may be more pulsatile or negligible under physiological conditions. This hemodynamic shift could contribute to chronic wall stress on the aneurysm dome in fetal-type cases. These distinct flow characteristics have important implications for aneurysm pathophysiology. Elevated WSS and concentrated inflow jets are known stimuli for aneurysm growth and wall degeneration in many locations [24]. The fPCOM configuration effectively creates a bifurcation aneurysm at the ICA-PCOM junction, which tends to incur more high-flow hemodynamic stress than a small sidewall PCOM aneurysm. Consistently, multiple studies have identified fPCOM anatomy as a risk factor for aneurysm development and rupture. A recent systematic review and meta-analysis found a significant association between fPCOM and PCOM aneurysm rupture [25]. In a single-center series using radiomic analysis, the presence of a fPCOM was associated with a 3-fold higher risk of rupture even after adjusting for size and shape parameters [26]. The presumed mechanism is that the increased flow and wall shear stress in the fetal variant accelerates aneurysm growth and destabilizes the wall [26]. Our hemodynamic results support this mechanism; the combination of high WSS, concentrated inflow, and low flow pulsatility in fPCOM aneurysms may promote continuous remodeling of the aneurysm wall, potentially making them more prone to rupture or recurrence. Tanaka et al. observed that simulated coil embolization left higher residual flow in fPCOM aneurysms, suggesting a propensity for incomplete occlusion and recanalization [10]. Combined, the hemodynamic profile of fPCOM aneurysms is one of persistent high-flow stress, which correlates with their higher rupture risk compared to aPCOM counterparts [25]. The distinct flow environment in fPCOM aneurysms has practical implications for therapy. In a large series of 480 PCOM aneurysms treated with coiling, Choi et al. reported an overall recurrence rate of about 33%, with a trend toward more frequent recanalization in fPCOM cases (37.8% vs. 26.9%) [8]. However, this difference was not significant after accounting for aneurysm size as well as other factors [8]. It is plausible that the robust inflow from a dominant fPCOM can promote coil compaction or residual filling, leading to higher re-treatment rates. Flow diversion has emerged as an alternative, particularly for wide-necked or recurrent aneurysms, but its efficacy in the PCOM location appears to be markedly influenced by fetal circulation anatomy. Flow-diverting stents (FD) rely on redirecting flow across the aneurysm neck. However, if a large-caliber fPCOM is supplying the posterior cerebral circulation, blood flow will continue through the PCOM and may maintain perfusion of the aneurysm. A multi-center study by Rinaldo et al. demonstrated that PCOM aneurysms in patients with a fPCOM circulation had significantly lower occlusion rates after flow diversion. Complete aneurysm occlusion at last follow-up was achieved in only 44% of fPCOM cases, compared to 82% in aPCOM [6]. Moreover, the median time to occlusion was longer in the fetal group (median 51 months vs. 6 months) [6]. Ten Brinck et al. also reported 0% occlusion in their series of fPCOM aneurysms treated with Pipline Embolization Device, (Medtronic, USA) [27]. Their review of the literature showed a pooled complete occlusion rate of only 31% for fPCOM aneurysms with FD, underscoring that two-thirds or more of these aneurysms fail to completely thrombose with standard flow diversion [27]. They propose that the underlying mechanism is hemodynamic. In the presence of a fetal-type PCOM, a high-flow conduit across the device may develop. As a result, blood flow from the ICA into the large PCOM may persist despite FD placement, which could in turn reduce the efficacy of flow diversion [27]. Given these considerations, management strategies for fPCOM aneurysms should be individualized and may favor adjunctive treatments. If flow diversion is considered at all, some authors suggest using devices with higher metal coverage. A recent simulation study comparing FD devices found that the newer high-coverage FD achieves greater flow reduction in a fPCOM aneurysm model, reducing sac velocity, high-flow volume, and WSS by roughly 50–80% [28]. In summary, recognizing a fPCOM circulation configuration is crucial in planning PCOM aneurysm treatment. The heightened flow and WSS in these aneurysms predispose them to rupture and pose challenges to durable endovascular occlusion.

Our findings, placed in context with the current literature, suggest that fPCOM aneurysms warrant aggressive management and careful follow-up. Coil embolization remains a first-line option for many, but recurrence is common and should be anticipated. FD, while an attractive minimally invasive solution for difficult aneurysms, have substantially lower efficacy in the presence of a fPCOM [6]. When faced with a fPCOM aneurysm, the treating team should consider adjunct strategies such as stent assisted coiling, combined FD and coiling or microsurgical clipping [27]. Tailoring the therapy in this manner, guided by hemodynamic insights, may improve occlusion rates and reduce the risk of re-hemorrhage, while safeguarding the important posterior circulation perforators supplied via the fPCOM. In addition to the therapeutic and follow-up implications of the hemodynamic differences observed between fPCOM and aPCOM bifurcation aneurysms, a crucial step toward clinical translation is the integration of these findings into routine practice. Individualized CFD analyses would need to become part of the clinical workflow. However, the generation and availability of CFD models requires considerable computational resources that are not typically available in standard hospital settings. A promising approach has been proposed by Temor et al., who developed and evaluated a pipeline for translating unstructured CFD data into structured DICOM datasets, thereby enabling visualization of flow dynamics within established PACS systems and DICOM viewers. Such an implementation could play a pivotal role in treatment planning, particularly when combined with simulations of different therapeutic strategies. Nevertheless, the work by Temor et al. has several limitations. The DICOM format was not originally designed for simulated data, and their encoding remains partly ad hoc. Regions of interest and voxel size selection was manual, introducing variability. Temporal resolution was limited by the viewer’s constraints, and the approach was tested only with a single radiographer using one specific system, limiting generalizability [29]. Future research should continue to integrate highly resolved patient-specific CFD analysis into treatment decision-making, seeing as understanding the flow environment can help predict which aneurysms are at highest risk of recurrence and may guide the development of improved devices or techniques for these challenging aneurysm subsets.

### Limitations

Several limitations of the study should be considered when interpreting the results. The comparatively small sample size of 14 cases limits the statistical significance and generalizability of the results. In addition, this is a retrospective analysis in which the selection of cases was dependent on the availability of suitable image data. This can lead to a selection bias. The methodology used is based on CFD. Although these are established methods, they only permit a simplified representation of the physiological conditions. The standardized inflow conditions disregard inter-individual differences, although intra-aneurysmal hemodynamics is known to be sensitive regarding the inflow condition [30]. But corresponding data was not available, and the effect is limited due to the relative comparison between the configurations. Nevertheless, future studies could profit from employing flow-rate–independent methods in the evaluation of hemodynamics [31]. The models used rigid vessel walls, meaning that pulsatile wall movements and their influence on hemodynamics were not included. However, this influence is considered to be small [32]. In this study, the modeling of fetal and adult PCOM flow scenarios is achieved by adjusting the outflow boundary conditions at the PCOM and subsequently at the distal bifurcation, without incorporating anatomical modifications of the vessel geometry. While prescribing flow rates independently of vessel caliber is not ideal, artificially modifying vessel geometry would be arbitrary and subjective. The imposed flow rates in the Pcom may yield locally non-physiological values, but these occur sufficiently distant from the aneurysm, which serves only as the region of interest for postprocessing. Furthermore, the categorization of outflow variations relies on estimated values, as no patient-specific flow measurements were available. To date, no studies quantify PCOM flow, particularly in fetal-type configurations, that could serve as a reliable ground truth for validation. Consequently, the applied flow distributions remain approximations and represent a potential source of uncertainty in the hemodynamic analyses. To generate valid data, future flow MRI measurements studies of anatomical PCOM variations are necessary.

## Conclusion

In paired, CFD of PCOM bifurcation aneurysms, fPCOM exhibited higher intra‑aneurysmal V_mean_, V_max_, AWSS, NIR, and ICI, with lower PI. In summary, these metrics delineate a high‑flow, concentrated‑jet, high‑shear hemodynamic environment in fPCOM compared with aPCOM subtypes, consistent with a possible distinct, subtype‑specific hemodynamic risk profile. To conclude, the anatomical variation of the PCOM has a impact on the hemodynamics of PCOM bifurcation aneurysms and delineates them into two distinct aneurysm subtypes. Recognition in advance of this pattern may inform risk stratification, endovascular treatment strategy selection, lowering the recurrence rates and improve the follow‑up planning in PCOM aneurysm care.

## Supplementary Information

Below is the link to the electronic supplementary material.

Supplementary Material 1 (PDF 8.01 MB)

## Data Availability

All data generated or analyzed in this study are included in this article and its supplementary file.
