# Correction to: Fetal-type posterior communicating artery increases hemodynamic stress in posterior communicating artery bifurcation aneurysms: a CFD-based analysis

**DOI:** 10.1007/s00234-025-03812-w

**Published:** 2025-10-14

**Authors:** Roland Schwab, Rebecca Janiszewski, Erelle Fuchs, Maximilian Thormann, Belal Neyazi, Vanessa Magdalena Swiatek, I. Erol Sandalcioglu, Philipp Berg, Daniel Behme, Samuel Voß, Janneck Stahl

**Affiliations:** 1 University Clinic for Neuroradiology, University Hospital Magdeburg, Magdeburg, Germany; 2 Research Campus STIMULATE, Otto-von-Guericke University Magdeburg, Magdeburg, Germany; 3 Department of Nuclear Medicine, Charité - University Medicine Berlin, Berlin, Germany; 4 Department of Radiology and Nuclear Medicine, University Hospital Magdeburg, Magdeburg, Germany; 5 Department of Neurosurgery, Otto-von-Guericke University Magdeburg, Magdeburg, Germany; 6 Research Campus STIMULATE, Otto-von-Guericke University Magdeburg, Magdeburg, Germany; 7 Department of Medical Engineering, Otto-von-Guericke University Magdeburg, Magdeburg, Germany; 8 Department of Fluid Dynamics and Technical Flows, Otto-von-Guericke University, Magdeburg, Magdeburg, Germany

**Correction to: Neuroradiology**

10.1007/s00234-025-03785-w

The original version of this article unfortunately contained a mistake. 

In the Abstract section, the first sentence under Background is incorrectly captured as; 

**Backround** The cs of bifurcation aneurysms in the posterior communicating arterflow characteristiy (PCOM) have rarely been studied.

The correct version should read as follows:

**Backround** The flow characteristics of bifurcation aneurysms in the posterior communicating artery (PCOM) have rarely been studied. 

In the Keywords section, the last two entries was incorrectly merge. Below is the incorrect keywords;

**Keywords** Aneurysm · Endovascular · Hemodynamics, PCOM 

The correct set of keywords should read as follows:

**Keywords** Aneurysm · Endovascular · Hemodynamics · PCOM 

The original article has been corrected.

