# Supplementary material for: Fetal-type posterior communicating artery increases hemodynamic stress in posterior communicating artery bifurcation aneurysms: a CFD-based analysis
Source: Neuroradiology. 2025 Sep 20;67(9):2471–81. doi: 10.1007/s00234-025-03785-w (PMC12546534; doi:10.1007/s00234-025-03785-w)
Supplement: Supplementary file 1 — Supplementary Material 1 (PDF 8.01 MB) [file 234_2025_3785_MOESM1_ESM.pdf]

Online supplemental data

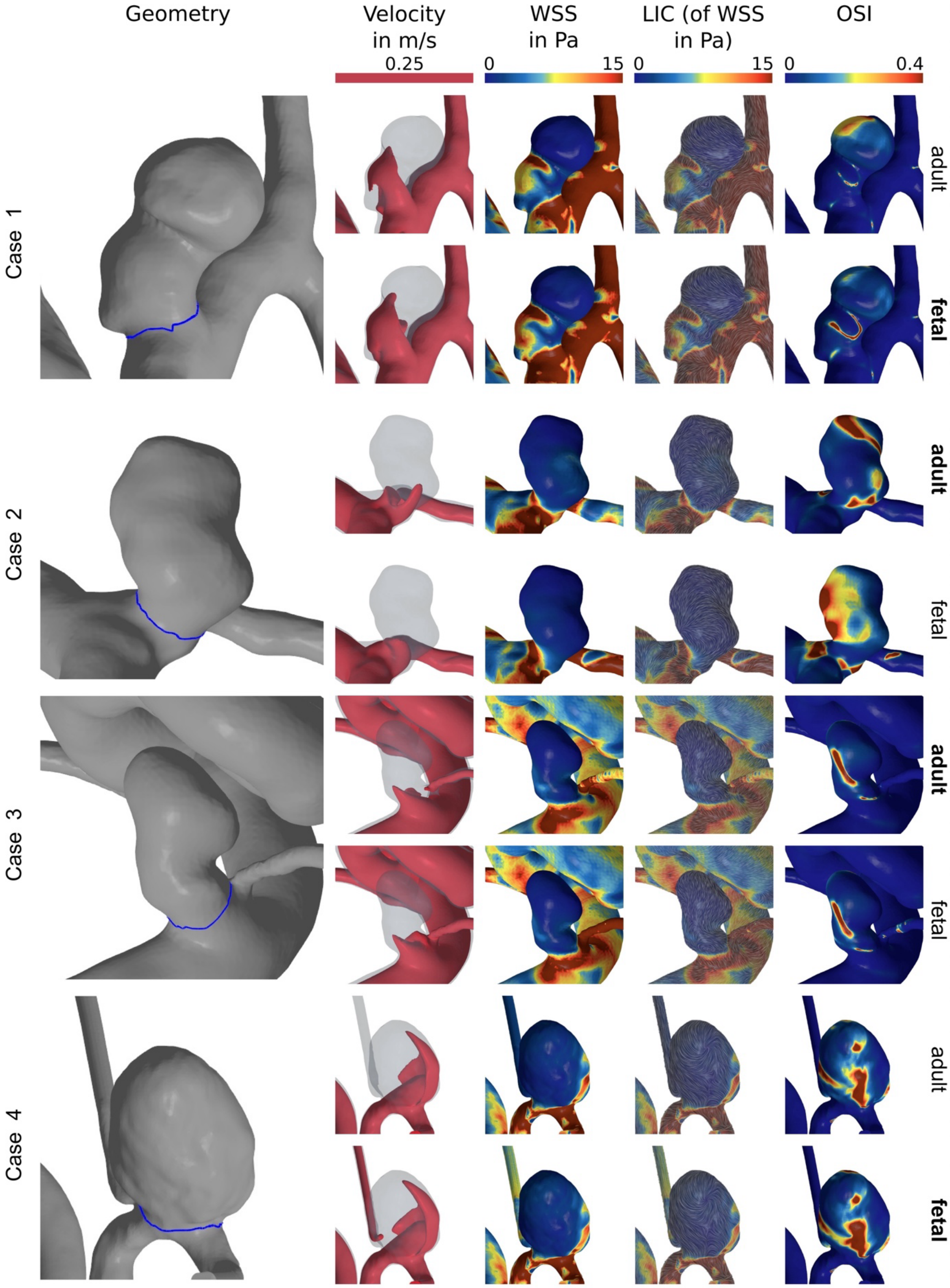

Supplementary Figure 1 Overview of the qualitative hemodynamic results for case 1-4. The patient-specific PCOM outflows are shown in bold and the artificially modeled outflow configurations in regular font.

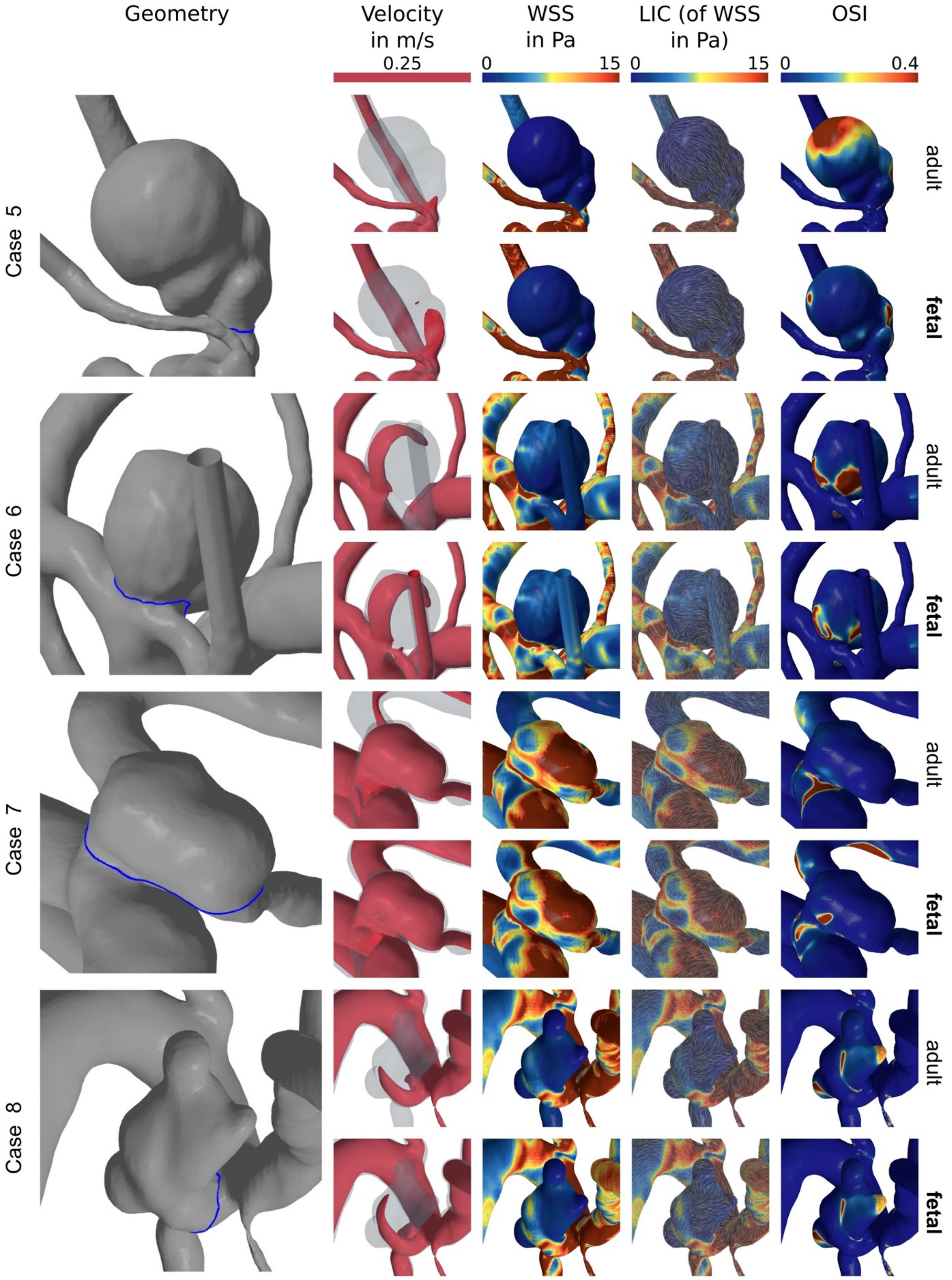

Supplementary Figure 2 Overview of the qualitative hemodynamic results for case 5-8. The patient-specific PCOM outflows are shown in bold and the artificially modeled outflow configurations in regular font.

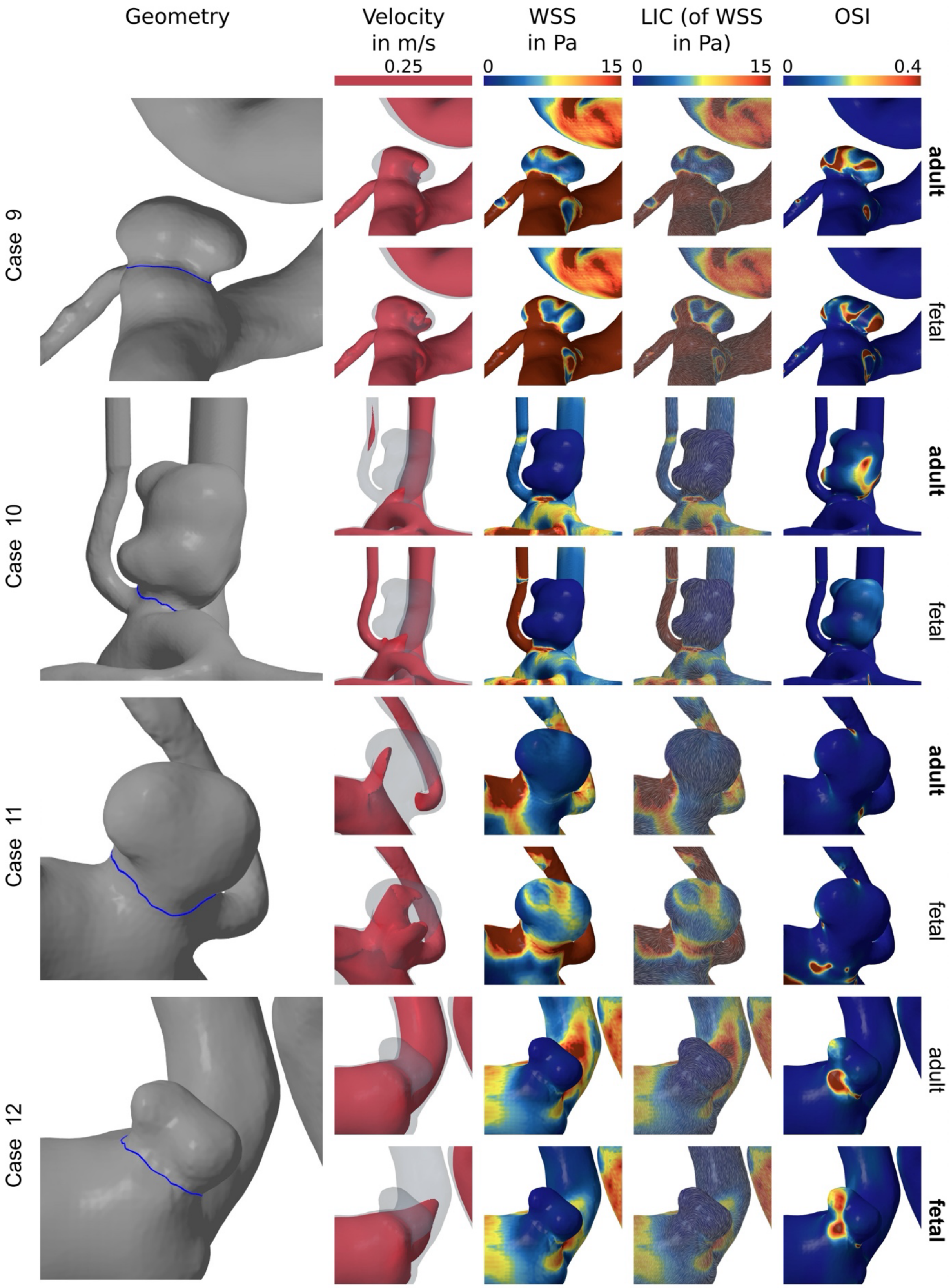

Supplementary Figure 3 Overview of the qualitative hemodynamic results for case 9-12. The patient-specific PCOM outflows are shown in bold and the artificially modeled outflow configurations in regular font.

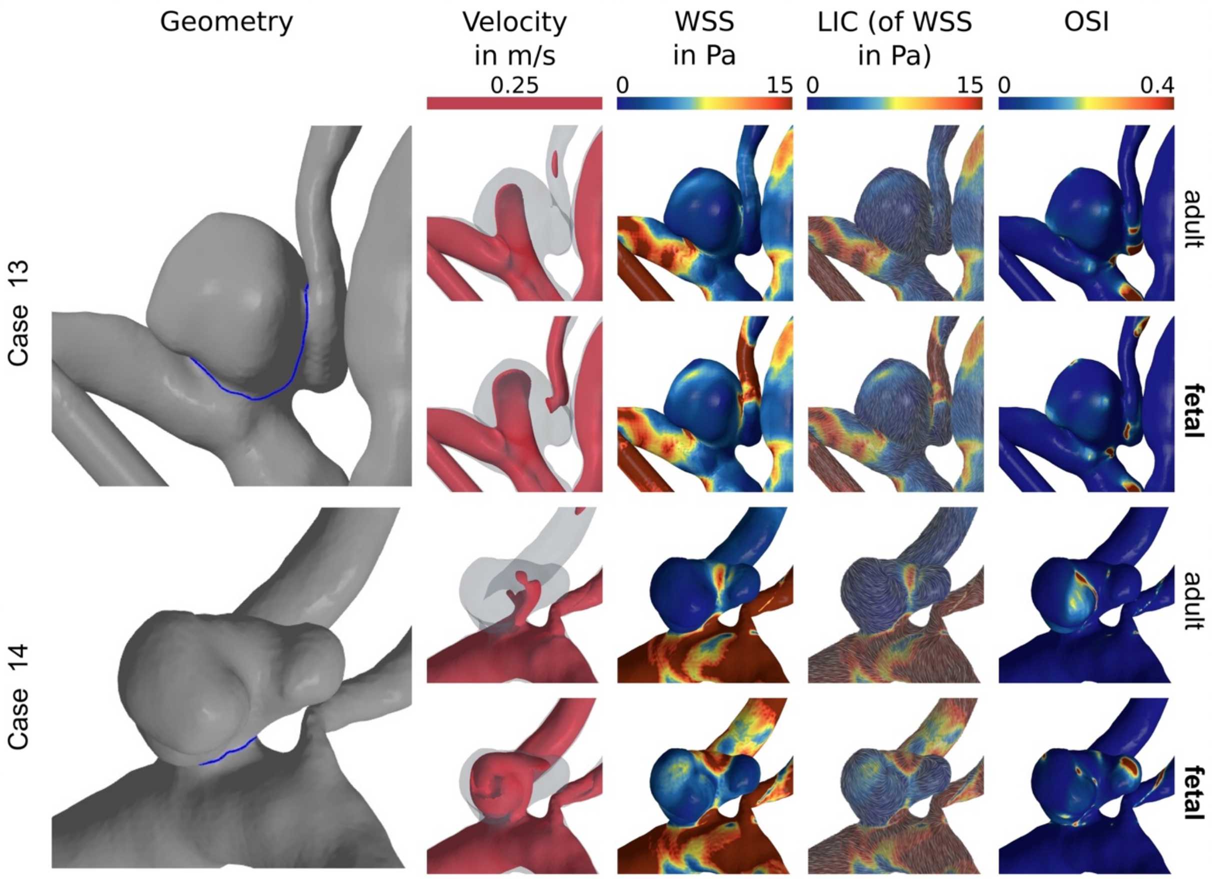

Supplementary Figure 4 Overview of the qualitative hemodynamic results for case 13-14. The patient-specific PCOM outflows are shown in bold and the artificially modeled outflow configurations in regular font.
